# A Skinfold Imitating a Pneumothorax

**DOI:** 10.7759/cureus.68654

**Published:** 2024-09-04

**Authors:** Amin Alayyan, Tarek Hammad, Salman Majeed

**Affiliations:** 1 Internal Medicine, Northampton General Hospital NHS Trust, Northampton, GBR; 2 Cardiology, Northampton General Hospital NHS Trust, Northampton, GBR

**Keywords:** skinfold, ct chest, chest x-ray, artifact, pseudo-pneumothorax, pneumothorax

## Abstract

We present the case of a male patient in his late 80s who presented with a fall with symptoms and signs of community-acquired pneumonia. Chest X-ray showed the suspicion of a left-sided pneumothorax. A CT of the chest subsequently ruled out the presence of a pneumothorax on the left side. The pseudo-pneumothorax on the chest X-ray was secondary to a skinfold. This case highlights how well a skinfold can mimic pneumothorax. Careful clinical and radiological examination with bedside lung ultrasound and/or CT of the chest can help differentiate true pneumothorax from pseudo-pneumothorax, provided the patient is hemodynamically stable. Our case highlights the importance of clinical examination, various imaging modalities, and confirmation of a diagnosis before proceeding to interventional procedures in the context of limited clinical suspicion of the differential.

## Introduction

Pneumothorax is the collapse of the lung when air accumulates inside the pleural space, which can happen spontaneously or from trauma. Spontaneous pneumothorax is further classified as primary (absence of an underlying lung disease) or secondary (presence of an underlying lung disease, such as chronic obstructive lung disease and pneumonia) [1,2].

Pneumothorax is a common thoracic pathology that can range in severity from completely asymptomatic to severe hemodynamic instability, resulting in cardiac arrest. Hemodynamically unstable patients with high clinical suspicion must be managed promptly with chest drain insertion to avoid loss of cardiac output [3]. In cases not requiring immediate intervention, confirming the diagnosis with plain film is usually straightforward, provided the clinician can identify the correct radiological features of a pneumothorax and rule out the false-positive causes that can mimic a pneumothorax. Certain mimics have been described in the literature that can imitate the appearance of a pneumothorax. These include bony artifacts, bedsheets, clothes, skinfolds, and primary lung pathologies such as emphysematous bullae, as well as pleural cysts [4]. Differentiating between a true and false pneumothorax is vital to avoid unnecessary procedures that could potentially result in detrimental complications [4,5].

## Case presentation

A male patient in his late 80s with a background of recurrent falls, dementia, ischemic heart disease, chronic kidney disease stage 3A, and psoriasis presented to the emergency department after he was found by the ambulance crew on the floor next to his bed. He was also found to be dyspneic and confused, unable to give a complete history. He required 3 L/minute of oxygen via a nasal cannula, and his saturation levels were 93%. His blood pressure was 102/54 mmHg, with a heart rate of 52 beats/minute and a respiratory rate of 24 breaths/minute, and he was afebrile.

Physical examination revealed course crackles on the right base but equal air entry on both bases. His heart sounds were normal. His peripheries were warm and well-perfused with a capillary refill of less than two seconds. Blood test results on admission are shown in Table 1.

**Table 1:** Blood test results.

Blood test (unit)	Result	Reference range
Haemoglobin (g/L)	119	130–170
White cell count (× 10⁹/L)	11.2	4.0–10.0
Neutrophil count (× 10⁹/L)	9.83	1.5–6.5
Lymphocyte count (× 10⁹/L)	0.76	1.1–3.5
Platelet count (× 10⁹/L)	316	150–400
International normalized ratio (INR ratio)	1.1	0.8–1.2
C-reactive protein (mg/L)	132	0–5
Estimated glomerular filtration rate (mL/minute)	59	90–120
Creatinine (µmol/L)	95	59–104
Sodium (mmol/L)	137	133–146
Potassium (mmol/L)	4.5	3.5–5.3
Albumin (g/L)	23	35–50
Corrected calcium (mmol/L)	2.48	2.20–2.60
Total bilirubin (µmol/L)	9	0–21
Alanine transaminase (IU/L)	10	5–41
Alkaline phosphatase (IU/L)	67	30–130

**Figure 1:**
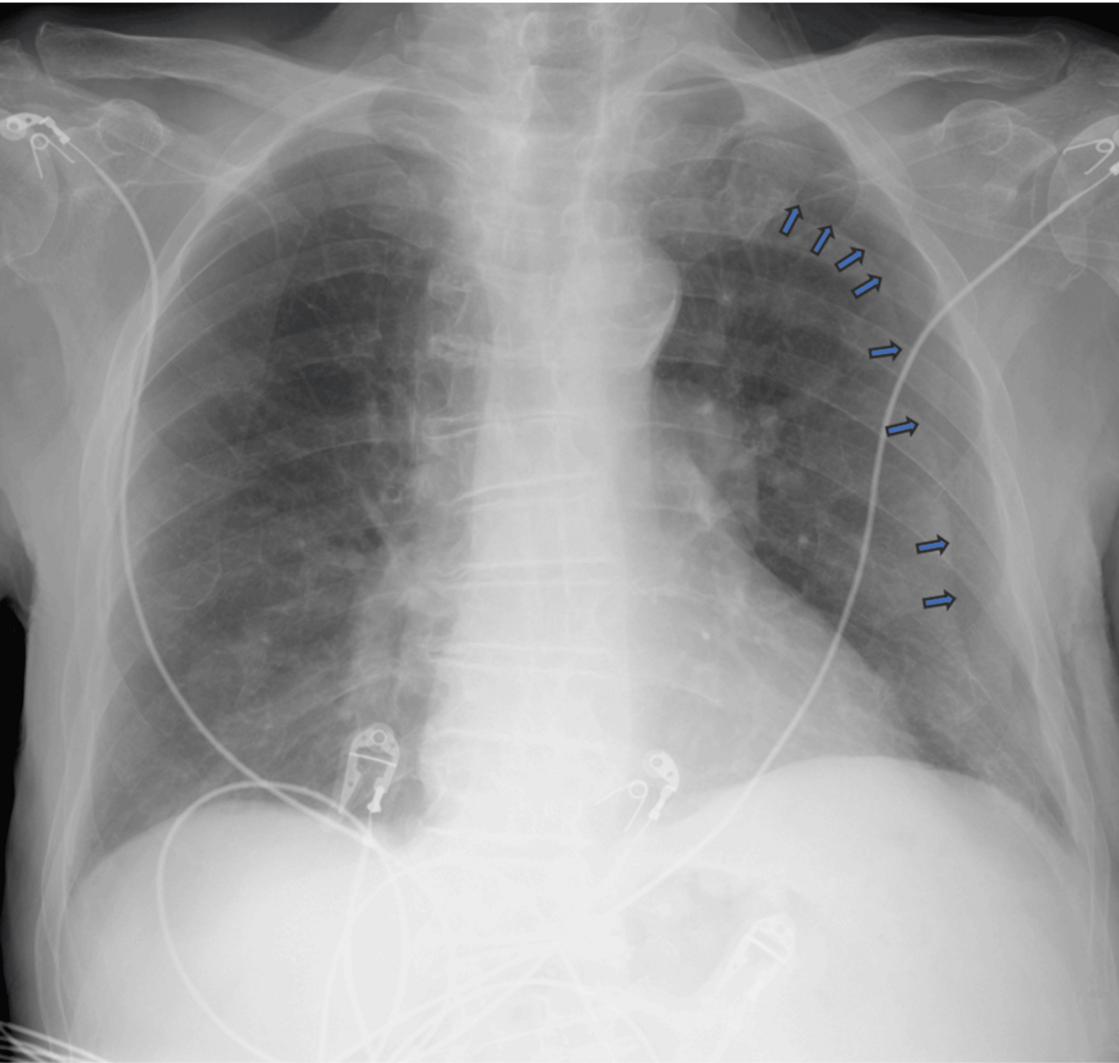
Anteroposterior chest X-ray showing a left skinfold, mimicking a left-sided pneumothorax. Blue arrows highlight the skinfold lining course starting as curved superiorly and becoming linear.

**Figure 2:**
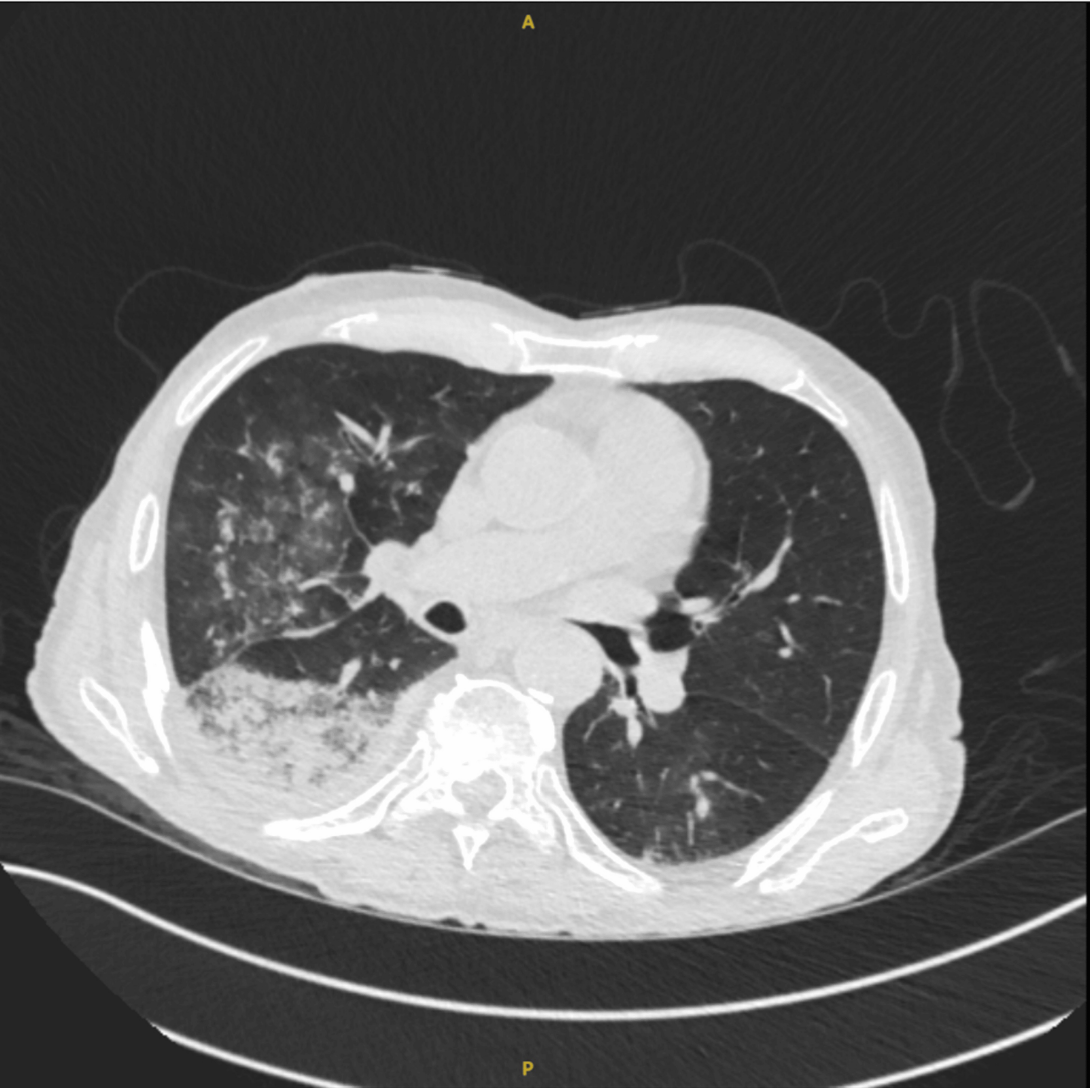
CT of the chest showing right lung parenchymal infective/inflammatory changes and confirming the absence of left-sided pneumothorax.

**Figure 3:**
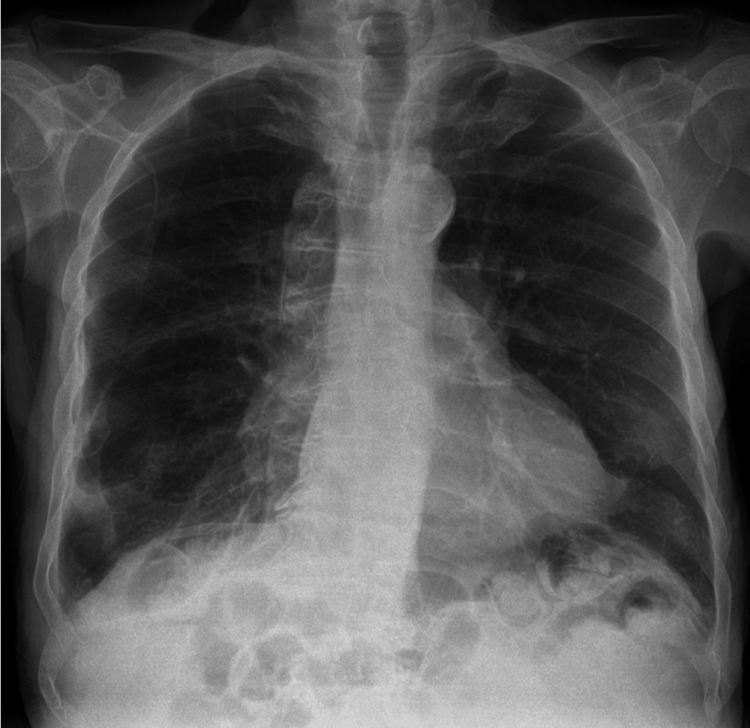
Posteroanterior chest X-ray not showing skinfold lining or pneumothorax.

Anteroposterior (AP) chest X-ray revealed right-sided very mild reticulonodular infiltrates which were presumed to be infective changes. However, it also showed a well-defined left hemithorax lining, raising suspicion of a left-sided pneumothorax (Figure 1). Following these findings, and a discussion with the on-call radiologist, who confirmed lung markings were visualized on both sides of the lining, a chest CT was performed which ruled out a pneumothorax but showed more extensive right lung parenchymal infective changes (Figure 2). The cause of the artifact was later concluded to be due to a skinfold. The patient was treated with intravenous piperacillin with tazobactam and oral clarithromycin. He was weaned from supplemental oxygen, his oxygen saturation levels improved, his C-reactive protein levels improved, and he was discharged from the hospital. He was clinically well and has been scheduled for routine follow-ups with his general practitioner post-discharge. Posteroanterior chest X-ray was later obtained post-discharge and showed no artifact (Figure 3).

## Discussion

This case shows how easily a skinfold can be mistaken for a pneumothorax if not appropriately reviewed by an expert eye. The superior aspect of the lining appears to be curvilinear before becoming straight as it is traced inferiorly (blue arrows in Figure 1). This gives the initial appearance that the lining resembles visceral pleural anatomy, which naturally follows the borders of the chest wall [6]. In addition, several case reports highlight the features of skinfolds as being linear throughout the lining, which differs from what is seen in this case [4,5]. This case can present a challenge in differentiating the skinfold from a pneumothorax.

Despite the above features making the image appear as a pneumothorax, many other radiological signs can help distinguish a skinfold from a true pneumothorax. The lining in the AP chest X-ray becomes ill-defined as it approaches the border of the chest wall and then completely disappears. Furthermore, lung markings can still be seen lateral to the lining in a patient with a skinfold, which is usually absent in a pneumothorax [3]. The absent lung markings would significantly increase the lucency lateral to the visceral pleura. Skinfolds can give the illusion of increased lucency lateral to the lining. Due to the increased thickness of skinfolds, the perceived image of the lung behind the skinfold appears darker compared to adjacent areas of the lung without any skinfolds. The difference in luminescence intensifies the margins of these areas, which causes the clinician to visualize the artifact lining; this is known as the Mach band effect [3,7].

This case also emphasizes the importance of careful clinical correlation and considering further diagnostic modalities to confirm the presence of a pneumothorax if there is a clinical suspicion. The literature provides cases where patients had detrimental complications secondary to thoracostomy tube insertion when chest X-ray findings mimicked pneumo-thoraces [4,8].

Increased emphasis is being put on chest CT and lung ultrasound for diagnosing pneumothorax if clinical assessment and plain film remain equivocal. Although CT is considered the gold standard for confirmation [3], lung ultrasound demonstrated a sensitivity and specificity of around 90% and 98%, respectively, in one systematic review and meta-analysis [9]. It also has the added benefit of being quick and cost-effective and not exposing patients to radiation [10].

## Conclusions

This was a challenging case of pseudo-pneumothorax secondary to a skinfold, and a lack of knowledge of confirming this could have resulted in the erroneous and harmful insertion of a chest drain. Skinfolds are one of the many imitators of pneumothorax and can be explained by the Mach band effect as to how the human eye can perceive the lining. The imaging in the case also showed the artifact to span toward the superior aspect of the left lung, which makes it difficult to differentiate it from an actual pneumothorax. Features that support the possibility of an artifact are poorly defined lining with sudden termination, the presence of lung markings, and the absence of increased lucency lateral to the lining. Knowing the causes of false pneumothorax and the findings that make pneumothorax more or less likely can help avoid unnecessary and risky interventions. Proper clinical assessment with training in bedside and advanced imaging modalities is crucial in confirming diagnosis.
